# Late Gadolinium Enhancement imaging using stack of stars and compressed sensing

**DOI:** 10.1186/1532-429X-13-S1-P162

**Published:** 2011-02-02

**Authors:** Ganesh Adluru, Liyong Chen, Seong-Eun Kim, Norman Hu, Kyle H Sabey, David A Bull, Eugene Kholmovski, Nassir Marrouche, Edward DiBella

**Affiliations:** 1 University of Utah, Salt Lake City, UT, USA

## Objective

To develop and test a 3D hybrid radial(stack of stars) acquisition scheme with compressed sensing(CS) reconstruction for high resolution late gadolinium enhancement(LGE) imaging of the left ventricle(LV) and the left atrium(LA).

## Background

LGE imaging is the gold standard for identifying viable tissue and fibrosis in the LV [1]. It has also been recently applied to imaging of the LA to identify regions of RF ablation [2] and fibrosis [3]. While a Cartesian acquisition scheme is commonly used, it can take a long time [1] and can be sensitive to motion leading to inconsistent image quality [3,4]. A radial acquisition scheme can be advantageous due to its robustness to motion and undersampling. Here a hybrid radial acquisition is combined with CS to improve LGE imaging efficiency.

## Methods

**Figure 1:**
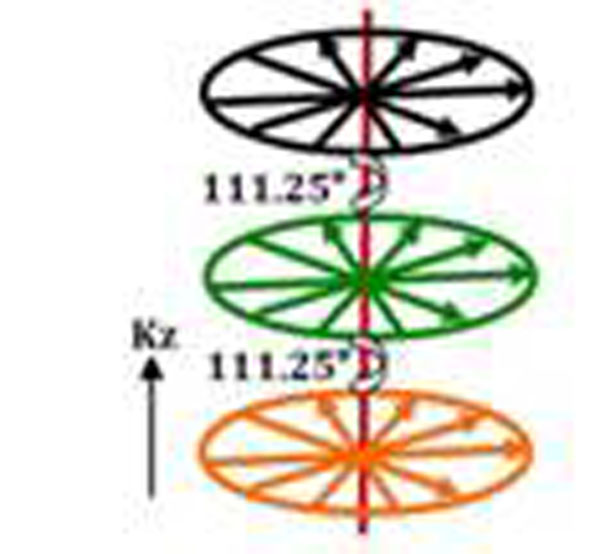
Schematic of kz-first hybrid radial lines after the inversion pulse are first acquired along kz before acquiring kx-ky. The kx-ky planes are color coded to illustrate consistency in the data.

A kz-first segmented acquisition was implemented on a Siemens 3T scanner. This scheme results in having consistent in-plane data with inconsistencies along kz leading to improved image quality as shown with simulation studies in [5]. As well, each plane is rotated by an angle based on golden ratio - Figure 1. This rotation helps exploit sparsity also along the z-dimension - a CS reconstruction with a 3D total variation constraint was used. Acquired data was first interpolated onto a 3D Cartesian grid and minimization of the cost function for CS was done with a gradient descent scheme.

The methods were tested for imaging the LV of three rabbits and for imaging the LA of three patients at least three months post-ablation. The scan parameters for the rabbit studies were TR=2.4 ms,TE=2.7ms,voxel size=0.9x0.9x2mm^3^,TI=300-350ms. For the patient studies the scan parameters were TR=3.4ms,TE=1.83ms,voxel size=1.25x1.25x2.5mm^3^,TI=300-400ms;36 slices with 144 rays in each plane were acquired in 1.25-2min.

## Results

**Figure 2:**
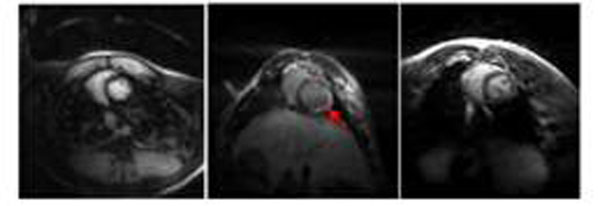
Results lf LGE imaging of the LV with proposed scheme. One representative slice from each of the three studies is shown. Arrow in the image points to enhancement.

**Figure 3:**
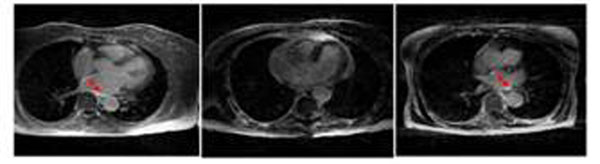
Results of LGE imaging of the LA. One representative slice from each of the three studies is shown. Arrows in the images point to enhancement.

Figure 2 shows the results from three rabbit studies. Enhancement in the lateral free wall corresponds to tissue necrosis from a ligation of a coronary artery. Axial slices of LA from three patient studies are shown in Figure 3. Enhancement corresponds to ablated regions in the atrium.

## Conclusion

The 3D stack of stars acquisition in conjunction with CS offers a promising alternative for rapid high resolution LGE imaging. More patient studies are required to validate the proposed framework.
